# Enrichment of Olive Oils with Natural Bioactive Compounds from Aromatic and Medicinal Herbs: Phytochemical Analysis and Antioxidant Potential

**DOI:** 10.3390/molecules29051141

**Published:** 2024-03-04

**Authors:** Paraskevi Yfanti, Polyxeni Lazaridou, Vasiliki Boti, Dimitra Douma, Marilena E. Lekka

**Affiliations:** 1 Department of Agriculture, University of Ioannina, 47150 Arta, Greece; 2 Institute of Environment and Sustainable Development, University Research Center of Ioannina (URCI), 45110 Ioannina, Greece; vboti@uoi.gr; 3 Department of Chemistry, University of Ioannina, 45110 Ioannina, Greece; p.lazaridou@uoi.gr; 4 Unit of Environmental, Organic and Biochemical High-Resolution Analysis Orbitrap-LC–MS, University of Ioannina, 45110 Ioannina, Greece; 5 Independent Researcher, 45110 Ioannina, Greece; dimitra@ddoumasons.com

**Keywords:** olive oil, phenolic compounds, antioxidant capacity, LC-LTQ/Orbitrap, GC-MS, aromatic herbs

## Abstract

Olive oil and herbs, two key components of the Mediterranean diet, are known for their beneficial effects on humans. In our study, we incorporated aromatic and medicinal herbs into local monovarietal olive oils via maceration procedures for enrichment. We identified the herbal-derived ingredients that migrate to olive oils and contribute positively to their total phenolic content and functional properties, such as radical scavenging activity. Thus, we characterized the essential oil composition of the aromatic herbs (GC-MS), and we determined the phenolic content and antioxidant capacity of the additives and the virgin olive oils before and after enrichment. The herbal phenolic compounds were analyzed by LC-LTQ/Orbitrap HRMS. We found that olive oils infused with *Origanum vulgare* ssp. *hirtum*, *Rosmarinus officinalis* and *Salvia triloba* obtained an increased phenolic content, by approximately 1.3 to 3.4 times, in comparison with the untreated ones. Infusion with *S. triloba* led to a significantly higher antioxidant capacity. Rosmarinic acid, as well as phenolic glucosides, identified in the aromatic herbs, were not incorporated into olive oils due to their high polarity. In contrast, phenolic aglycones and diterpenes from *R. officinalis* and *S. triloba* migrated to the enriched olive oils, leading to a significant increase in their phenolic content and to an improvement in their free radical scavenging capacity.

## 1. Introduction

Olive oil and aromatic herbs are important components of the Mediterranean diet, one of the healthiest diets worldwide [1]. Both have beneficial effects on human health, attributed mainly to their bioactive plant secondary metabolites. Olive oil comprises a saponified fraction (97–99%), consisting mainly of glyceryl fatty acid esters (mainly oleic acid) and a small non-saponified one (1–3%), containing bioactive compounds that affect the olive oil shelf life and its organoleptic properties and provide health benefits. In fact, extra virgin olive oil has been characterized as a functional food with high nutraceutical value [2]. On the other hand, aromatic plants, which are widely distributed in the Mediterranean region, constitute a specific category, producing essential oils. These compounds provide the characteristic odor and taste to the plant. Aromatic plants with medicinal properties are also known as medicinal and aromatic plants. Some species are used as herbs for flavoring foods but also as natural preservatives in the food industry due to their antimicrobial and antioxidant properties. The utilization of extra virgin olive oil and aromatic culinary herbs has been associated, among others, with lower mortality rates from cardiovascular disease, lower cancer indices due to their chemopreventive activity and a reduced risk from neurodegenerative diseases [3]. The enrichment of olive oils with aromatic herbs, which is a traditional practice in the Mediterranean region, has had significant impact in the market in recent years. Except for the improvement of organoleptic properties, the addition of aromatic herbs is expected to have a positive effect on the nutritional value of the enriched olive oils, upgrading their biofunctional properties, mainly attributed to phenolic compounds [4]. These belong to one of the main classes of secondary metabolites, which provide protection to the plant itself [5] by contributing to their adaptation under abiotic stress conditions and providing protection from biotic agents. These compounds are characterized by the presence of at least one phenolic hydroxyl group in their molecule. Their beneficial value in the human’s body is mainly attributed to their ability to protect cells (or tissues) from oxidative stress damages. According to our best knowledge, little is known about the migration of phenolic compounds from aromatic plants to olive oils during the enrichment and enhancement of their biofunctional properties. The aim of this study was to identify the ingredients of selected aromatic herbs that contribute to an upgraded phenolic content and antioxidant capacity of the enriched olive oils. For this purpose, we incorporated selected aromatic herbs into monovarietal olive oils from the region of Epirus, Greece and determined the phenolic content and antioxidant capacity of the additives and the olive oils before and after the enrichment. In addition, we proceeded to the characterization of phenolic compounds migrating from plants to the olive oils.

## 2. Results

### 2.1. Essential Oil Composition

The results of the GC-MS essential oil analysis are presented in Table 1 (Figure S1). In total, 24, 66 and 48 compounds were identified in *O. vulgare* ssp. *hirtum*, *R. officinalis* and *S. triloba* essential oils, respectively. From the fraction of monoterpenes, which were predominant (88.2–97.8%) in all the analyzed essential oil samples, the oxygenated compounds (63.7–92.7%) prevailed. Sesquiterpenes comprised a small portion (1.8–8.5%), while only one diterpene (manool 0.9%) was present in *S. triloba* essential oil. Carvacrol was the main constituent in *O. vulgare* ssp. *hirtum* (86.4%) essential oil, while the other monoterpenic phenol thymol comprised a small portion (4.6%). The oxygenated monoterpenic compound 1,8-cineole (44.2%) was the most abundant in *S. triloba* essential oil. The oxygenated monoterpenes camphor (13.2%), borneol (11.7%) and verbenone (11.5%) and the monoterpenic hydrocarbon α-pinene (12.3%) were found to be dominant in *R. officinalis*.

### 2.2. Total Phenolic Content

**Figure 1:**
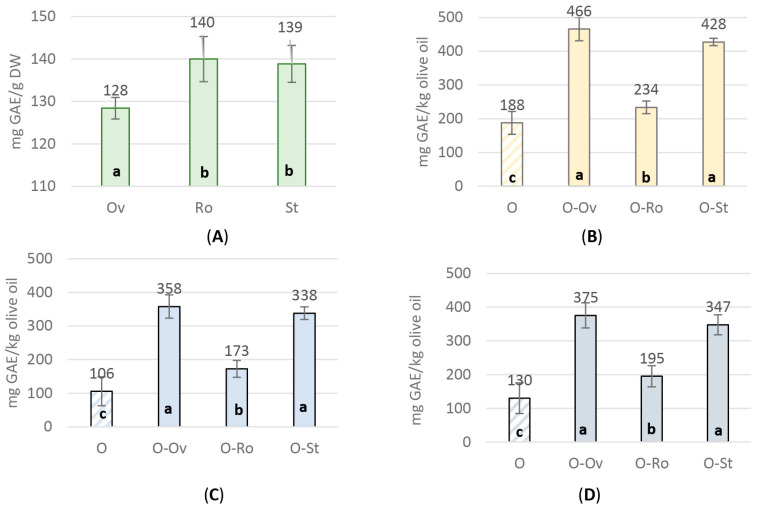
Total phenolic content of (**A**) aromatic plants MeOH extracts, and the hydro-methanolic extracts [MeOH-H_2_O (80:20, *v*/*v*)] of the untreated and infused olive oils; (**Β**) “Lianoelia Prevezas”; (**C**) “Konservoelia Artas”; and (**D**) “Lianoelia Kerkyras” olive oils. O: olive oil, Ov: *O. vulgare* ssp. *hirtum*, Ro: *R. officinalis*, St: *S. triloba*. a: statistical difference from; b: statistical difference from; c: statistical difference from. Values are expressed as means ± SD of four individual experiments. Means with different letter indications are significantly different (Duncan’s *p* < 0.05).

The total phenolic compounds of the tested aromatic plants (Figure 1A) ranged from 128.40 ± 0.89 to 139.98 ± 1.87 mg GAE g^−1^ DW. *R. officinalis* and *S. triloba* showed a significantly higher yield in phenolic content in comparison to *O. vulgare* ssp. *hirtum*. Concerning the three types of olive oils used in the experiment, the values of the total phenolic compounds varied between 105.84 ± 15.26 and 187.85 ± 18.53 mg GAE g^−1^ DW. Higher values were noticed for “Lianoelia Prevezas” (Figure 1B) compared to “Konservoelia Artas” (Figure 1C) and “Lianoelia Kerkyras” (Figure 1D). It is noteworthy that the infusion with aromatic plants significantly enhanced their content in polyphenols from 233.65 ± 18.65 to 427.50 ± 10.78 mg GAE Kg^−1^ for “Lianoelia Prevezas”, 172.68 ± 12.58 to 357.89 ± 13.66 mg GAE Kg^−1^ for “Konservoelia Artas” and 194.95 ± 15.61 to 375.40 ± 18.67 mg GAE Kg^−1^ for “Lianoelia Kerkyras” compared to the relevant untreated control olive oils.

### 2.3. Profile of Phenolic Compounds

The identified phenolic compounds in the MeOH extracts of the selected aromatic plants and the relevant compounds detected in the hydromethanolic extracts [MeOH-H_2_O (80:20, *v*/*v*)] of the enriched olive oils are shown in Table 2, Table 3 and Table 4 (Figure S2). For the majority of the compounds, the identification was based on the accurate mass determination of their deprotonated molecular ions [Μ − H]^−^ (with an exception for carnosol, naringenin [M + H]^+^) and their fragmentation patterns. The analysis of *O*. *vulgare* ssp. *hirtum* MeOH extract (Table 2) revealed the presence of two phenolic acids (salicylic acid, rosmarinic acid) and a phenolic acid glucoside (caffeic acid hexoside), five flavonoid glycosides (luteolin-6-C-glucoside, luteolin-6,8-di-c-hexose, apigenin 8-C-glucoside, apigenin 6,8-di-C-glucoside, eriodictyol 7-O-glucoside), seven flavonoid aglycones (luteolin, apigenin, acacetin, eriodictyol, naringenin, taxifolin, dihydrokaempferol) and the polyphenol salvianolic acid B. From these compounds, only the seven flavonoid aglycones and salicylic acid were detected in the hydromethanolic extracts of the three enriched olive oils. 

**Figure 2:**
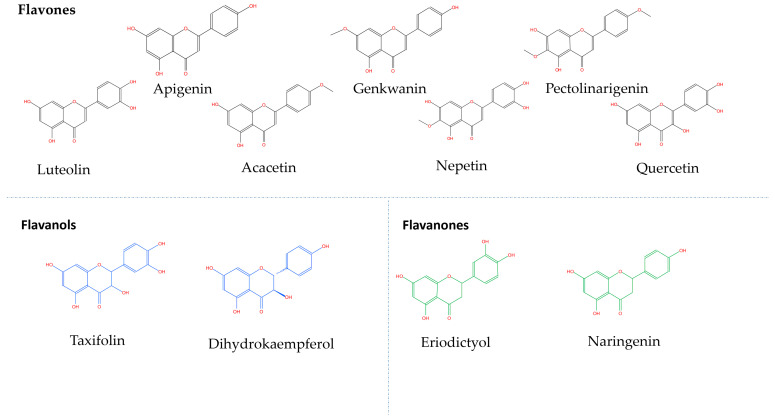
Phenolic aglycons migrating from the aromatic herbs to the olive oils analyzed by LC-LTQ/Orbitrap HRMS.

**Figure 3:**
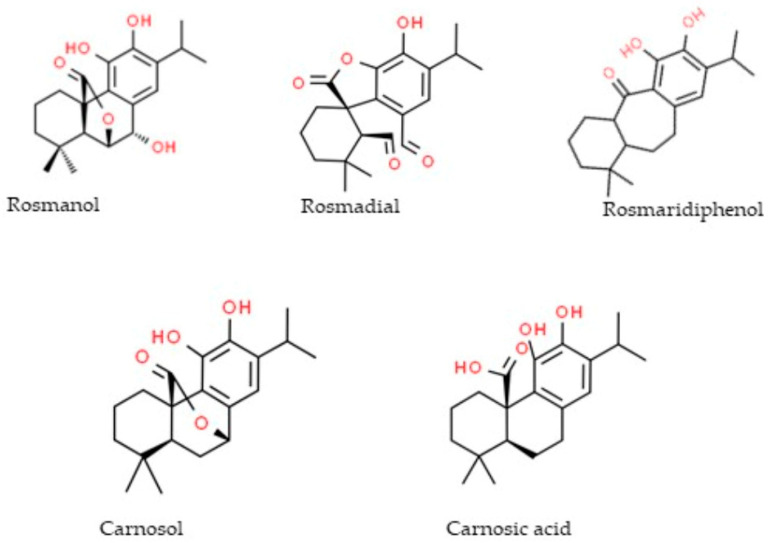
Diterpenic phenols migrating from the aromatic herbs to the olive oils analyzed by LC-LTQ/Orbitrap HRMS.

**Figure 4:**
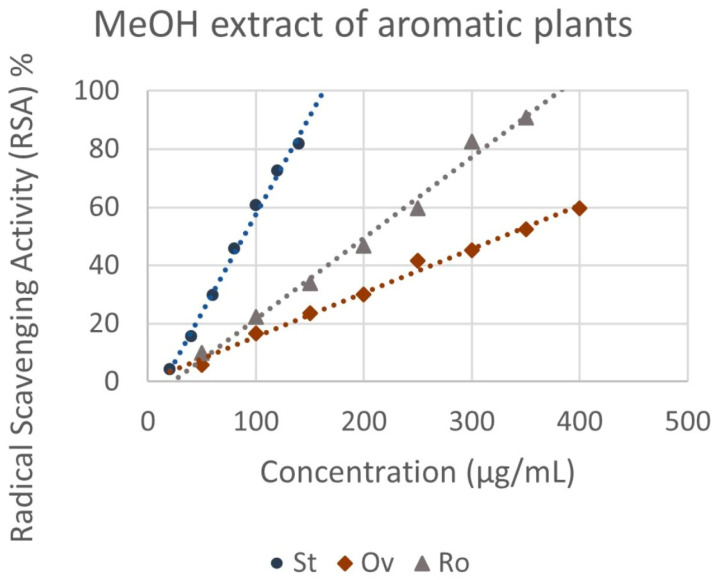
Antioxidant capacity of the methanolic extract obtained by the selected aromatic plants St: *S. triloba*, Ov: *O. vulgare* ssp. *hirtum*, Ro: *R. officinalis*, as assessed by DPPH assay. Values are expressed as means of four experiments.

Fourteen phenolic compounds were detected in the *R. officinalis* methanol extract (Table 3). Two of them belonged to the class of phenolic acids (rosmarinic acid, caffeic acid), two were flavonoid glycosides (hesperidin, hispidulin-7-glucoside), three were flavonoid aglycones (apigenin, pectolinarigenin, genkwanin) and seven were diterpenic phenols (rosmanol, hydroxyrosmanol, epirosmanol methyl ether, rosmadial, rosmaridiphenol, carnosol, carnosic acid). Ten of them, in particular the flavonoid aglycones and the diterpenic phenols, were detected in the hydromethanolic extracts of the *R. officinalis*-aromatized olive oils. A total of eighteen phenolic compounds were identified in the *S. triloba* extract (Table 4): three phenolic acids (vanillic acid, caffeic acid, rosmarinic acid), three flavonoid glycosides (nepetrin, luteolin-7-O-glucoside, luteolin-3-O-glucuronide), four flavonoid aglycones (nepetrin, apigenin, pectolinarigenin, quercetin), seven phenolic diterpenes (rosmanol, hydroxyrosmanol, rosmanol methyl ether, rosmadial, rosmaridiphenol, carnosol, carnosic acid) and one cyclobutane lignan (sagerinic acid). The identified flavonoid aglycones and the diterpenic phenols were also detected in the extract obtained by the *S. triloba*-enriched olive oils. For better observation of the results, the phenolic compounds that migrate from the plant to the olive oil are marked in gray (Figure 2, Table 2, Table 3 and Table 4). The chemical structure of compounds that migrated in the enriched olive oils from the aromatic herbs belongs mainly to the classes of flavones, flavanols and flavanones (Figure 3) and to phenolic diterpenes, which are shown in Figure 4.

### 2.4. Antioxidant Activity

**Figure 5:**
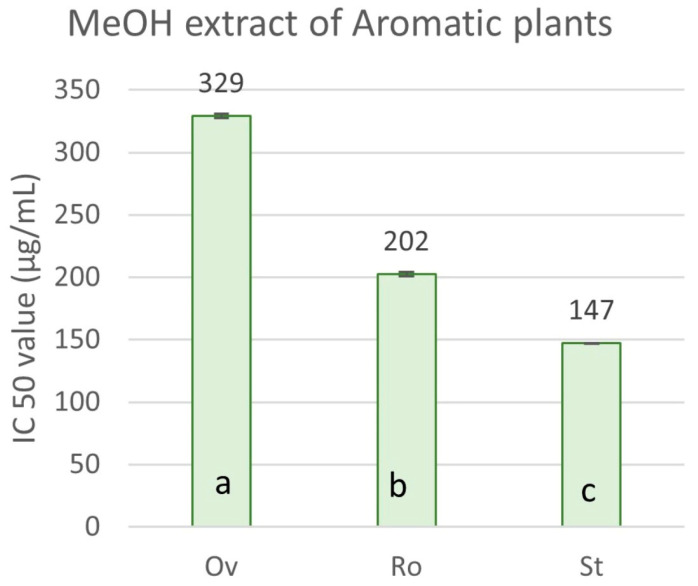
IC_50_ values of the selected aromatic plant species for radical scavenging. Ov: *O. vulgare* ssp. *hirtum*, Ro: *R. officinalis*, St: *S. triloba*, methanolic extracts. Values are expressed as mean ± SD of four experiments. Means with different letter indications are significantly different (Duncan’s test, *p* < 0.05).

Regarding the radical scavenging activity of the crude methanol extracts and the essential oils isolated from the aromatic plants, the results present significant variations (Figure 4). The concentration required to produce a 50% inhibition of the free radical (IC_50_) ranged from 147.09 ± 6.39 μg mL^−1^ to 328.93 ± 3.50 μg mL^−1^ for the ΜeOH extracts (Figure 5). *S. triloba* extract expressed the highest antioxidant activity (147.09 ± 6.39 μg mL^−1^), followed by *R. officinalis* (202.29 ± 0.57 μg mL^−1^), while *O. vulgare* ssp. *hirtum* (328.93 ± 3.50 μg mL^−1^) showed the lowest one. In contrast, the essential oils from *O. vulgare* ssp. *hirtum* (220.59 ± 4.03 μg mL^−1^) showed much higher scavenging activity for the DPPH free radical compared to *S. triloba* and *R. officinalis* essential oils, which showed a very weak capacity (8.90 and 10.30% RSA, respectively, at 1200 μg mL^−1^). The antioxidant activity index (AAI) for *O. vulgare* ssp. *hirtum*, *S. triloba* and *R. officinalis* methanol extracts was 0.12, 0.20 and 0.27, respectively, and 0.18 for *O. vulgare* ssp. *hirtum* essential oil.

**Figure 6:**
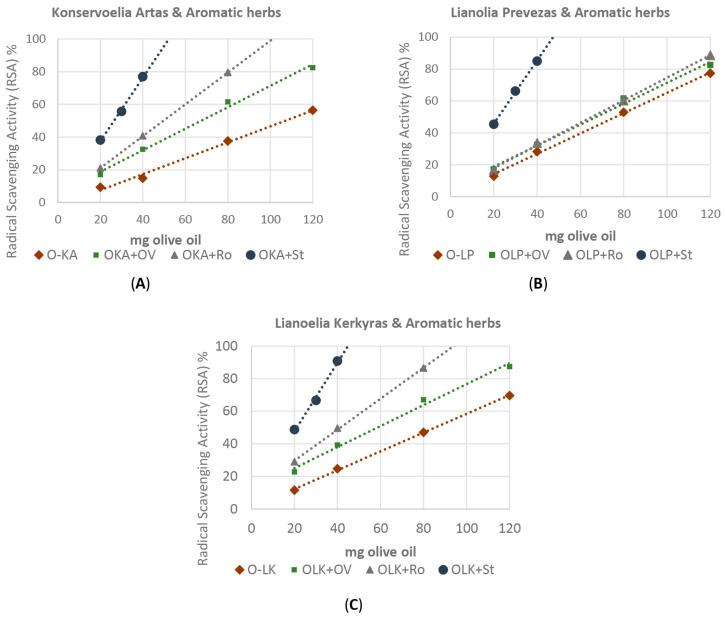
Antioxidant activity of the untreated and infused (**A**) O-LP: “Lianoelia Prevezas”; (**B**) O-KA: “Konservoelia Artas”; (**C**) O-LK: “Lianoelia Kerkyras” olive oil as assessed by DPPH assay. Ov: *O. vulgare* ssp. *hirtum*, Cc: Ro: *R. officinalis*. St: *S. triloba*. Values are expressed as means of four individual experiments. The analysis was performed by the DPPH assay.

According to the results of the DPPH assay, the antioxidant activity of the untreated and enriched extra virgin olive oils ranged from 132.6 ± 2.61 to 185.8 ± 4.1 and 167.3 ± 3.2 to 672.6 ± 22.2 mM gallic acid Kg^−1^ olive oil for the three olive oils, respectively (Table 5). All the aromatic plants used in the experiment were found to enhance the scavenging radical activity of the “Konservoelia Artas” (Figure 6A), “Lianoelia Prevezas” (Figure 6B) and “Lianoelia Kerkyras” (Figure 6C) olive oils. It is noted that the three infused olive oils differed in their antioxidant capacity. In particular, it was found that while *O. vulgare* ssp. *hirtum* enhanced the antioxidant capacity of the enriched olive oils, *R. officinalis* was relatively more effective, but the infused olive oils with *S. triloba* exhibited the highest antioxidant capacity.

## 3. Discussion

Foods, which, besides their basic ingredients and initial nutritional value, contain components of natural origin, providing health-beneficial properties to consumers and well-being, are characterized as biofunctional [10]. Olive oil is characterized as functional food due to its biofunctional chemical composition. Spices and various aromatic herbs are widely used as flavoring agents in olive oil. Aromatization enhances the flavor of the enriched olive oil, increases its qualitative characteristics and expands its shelf-life. Besides the traditional aromatization of olive oil with aromatic herbs in the Mediterranean region, infused olive oils have been more frequently available on the market during the last few years. Most studies deal with the effects of the additives on the sensory characteristics of the enriched olive oils and the extension of their self-life during storage [8,11,12,13,14,15].

The traditional procedure (maceration) for flavoring olive oils is to add the dry, ground aromatic herb into the olive oil and to keep it at room temperature. The mixture is frequently agitated in order to facilitate the diffusion of the compounds, while the flavored olive oil is obtained after filtration [12,16]. Another practice is to first isolate the essential oil from the aromatic plant and to subsequently incorporate it into the olive oil [16]. In this case, olive oil is enriched only with the plant volatile compounds, and the advantage is that there is no need to separate the plant material from the olive oil. Another proposed practice for flavoring is the addition of the plant material at the olive mills during the malaxation extraction process [12,16]. The monovarietal olive oil samples used in this study were obtained from cultivated olive varieties of the region of Epirus, Greece from the production of 2022. Two samples belong to recognized geographical indication (PGI) varieties: “Lianoelia Prevezas”, which is used to produce extra virgin olive oil, and “Konservoelia Artas”, which is an edible olive cultivar. The “Konservoelia Artas” olive oil is produced from olives that do not fit to the market standards as edibles. The third sample of olive oil was obtained from the old Venetian origin variety “Lianoelia Kerkyras” grown in the region of Parga. In our study, infused olive oils with the selected aromatic herbs *O. vulgare* ssp. *hirtum, R. officinalis* and *S. triloba* were prepared through maceration. Studies on the migration of plant tissue ingredients to olive oils, enhancing their biofunctional potential, are limited [14]. The aromatic herbs’ bioactive constituents belong to the plant’s secondary metabolites. Their chemical composition depends not only on the plant species that produces them but also on other factors, including the environmental conditions during plant growing, the stage of harvest and the drying and storage conditions. For this reason, we initially conducted phytochemical analysis of the plant material used in the present study. The selected aromatic plants with medicinal properties (Greek oregano, rosemary, Greek sage) are commonly used for food flavoring in the Mediterranean cuisine and are also used traditionally for their healing properties. As known, the bioactivity and medicinal properties of aromatic herbs are mainly attributed to the production of phenolic compounds [17] and volatile essential oil ingredients [18], which mainly belong to mono- and sesquiterpenes. The therapeutic value of the phenolic compounds is mainly attributed to their ability to protect from oxidative stress damage by different mechanisms due to the presence of at least one phenolic hydroxyl group [3].

First, we analyzed the methanol extracts of aromatic plants and we found higher levels in *S. triloba* and *R. officinalis* compared to *O. vulgare* ssp. *hirtum.* Although *S. triloba* και *R. officinalis* extracts presented an almost equal content of phenolic compounds, *S. triloba* possessed a higher antioxidant activity. This might be due to the structure of its phenolic compounds [19] or even due to synergistic or antagonistic effects with the ingredients of the methanol extract [20]. GC-MS analysis of the essential oils revealed that, in *O. vulgare*, ssp. *hirtum* and *S. triloba* carvacrol and 1,8-cineole were most abundant. The monoterpenes camphor, α-pinene, borneol and verbenone were at almost equal percentages in *R.* officinalis essential oil. The high content in carvacrol (>80%), which is known to possess a significant free radical scavenging activity [21], contributes to the reactivity of *O. vulgare* ssp. *hirtum* essential oil toward the DPPH radical. On the other hand, *S. triloba* and *R. officinalis* essential oils showed a much weaker antioxidant activity. These findings indicate the presence of nonvolatile compounds with high antioxidant activities in the methanol extracts of *S. triloba* and *R. officinalis*. For the evaluation of our data, we used the antioxidant activity index (AAI) proposed by Scherer and Godoy (2009) [22] to avoid fluctuations in the expression of the results due to different reaction conditions used by researchers. According to this index-based classification, the antioxidant activities of Origanum, Salvia and Rosmarinus methanol extracts, as well as that of Origanum essential oil, were characterized as moderate.

Regarding the hydromethanolic extracts of the infused olive oils, our results showed that all the selected aromatic herbs enhanced the phenolic content and improved the antioxidant capacity of all the infused olive oils. In particular, the total phenols were increased by approximately 2.5 to 3.4 times in Origanum and 2.3 to 3.2 times in sage-flavored olive oils, while, with rosemary, where the impact was weaker, the increase was about 1.3–1.6 times.

The greatest increase in the phenolic content of infused oils was observed for those with lower levels of phenolic compounds. Indeed, while the methanol extracts of *R. officinalis* and *S. triloba* presented a higher total phenolic content compared to the respective *O. vulgare* ssp. *hirtum*, we did not observe an equivalent increase in the hydromethanolic extracts of the relevant enriched olive oils. This could be due to a difference in the polarity of phenolic compounds, depending upon the number of hydroxyl groups attached to the aromatic ring [23], that influenced their solubility both in the olive oil as well as in the hydromethanolic mixtures used for their recovery from the infused olive oils.

However, it was remarkable that mainly the *S. triloba*-enriched olive oils and, subsequently, *R. officinalis*, presented significantly higher antioxidant activity compared to the *O. vulgare* ssp. *hirtum*-enriched ones. It is mentioned that the determination of the radical scavenging activity toward DPPH was conducted on the olive oil per se, and not on the revealed hydromethanolic extract, meaning that the sample contained all the ingredients of the enriched olive oils.

The above-mentioned results indicate that the aromatic herbs *S. triloba* and *R. officinalis* contain less polar phenolic ingredients that do not migrate to the hydromethanolic phase during the extraction of the infused oils (where the determination of total phenolic content takes place), but are present in the crude infused olive oils.

These observations are in consistency with the results of the LC-MS analysis used for the identification of the phenolic compounds. For example, rosmarinic acid, a polar phenolic acid compound with a high DPPH free radical scavenging activity [24], was identified in all the methanol extracts of the selected herbs used in this study, but it was not detected in the enriched olive oils. In contrast, salicylic acid, an *O. vulgare* ssp. *hirtum* phenolic acid with moderate polarity, migrated into the infused olive oils, enhancing their phenolic content. None of the phenolic acid glucosides were extracted into the olive oils. On the contrary, all the phenolic aglycones identified in the extracts of the aromatic herbs utilized, as well as the phenolic diterpenes present in *R. officinalis* και *S. triloba*, significantly increased the phenolic content of the enriched olive oils and contributed to their radical scavenging activity.

The phenolic compounds identified by LC-MS belong to the classes of phenolic acids, flavonoids and phenolic diterpenes. Bibliographic data suggest that specific phenolic compounds that have been established to migrate from the plant material to the olive oil may exhibit additional biofunctional properties, apart from their antioxidant activity. Reports mention the anti-inflammatory and anti-tumor properties of the flavones apigenin [25], genkwanin [26], luteolin [27], the flavanone eriodictyol [28], the flavanonol taxifolin [29], the flavonol quercetin [30], the phenolic diterpenes carnosol and carnosic acid [31] and rosmanol [32]. Among other beneficial properties, luteolin also has anti-diabetic, anti-hypertensive, anti-asthmatic and anti-viral properties [27]. Several studies demonstrate the beneficial effect of genkwanin against many diseases, including cardiometabolic diseases, type 2 diabetes and neurodegenerative disorders. Taxifolin also shows antimicrobial, cardiovascular hepatoprotective, anti-Alzheimer and antiangiogenic properties [29]. Naringenin has also been mentioned to possess antidiabetic, antibacterial, gastroprotective, immunomodulator, cardioprotective, nephroprotective and neuroprotective effects [33]. The bioactive flavonol quercetin exerts a variety of health-beneficial effects, such as antihypertensive, anti-hypercholesterolemic and anti-atherosclerotic, as well as neuroprotective, antibacterial, antiviral and antiallergic activities, and provides protection against cardiovascular diseases [30].

## 4. Materials and Methods

### 4.1. Reagents and Solvents

Water and acetonitrile (LC-MS grade) were purchased by Fisher Scientific (Leicester, UK). Formic acid (FA), 98–100% purity, was obtained from Merck (Darmstadt, Germany). DPPH, anhydrous sodium sulphate, gallic acid and methanol were obtained from Sigma-Aldrich (Steinheim, Germany). Ascorbic acid and Folin–Ciocalteu reagent were purchased from BioChemica (Sauerlach, Germany) and Supelco, (Bellefonte, PA, USA), respectively.

### 4.2. Plant Material and Olive Oils

The aromatic plants were collected from the region of Epirus, Greece. The aerial part of *Origanum vulgare* ssp. *hirtum* was collected at the flowering stage (municipality of Ziros, Preveza, Greece) and *Rosmarinus officinalis* shoots at the end of the autumn flowering period, (municipality of Arta, Arta, Greece), while shoots of *Salvia triloba* were collected after the fruiting period (municipality of Hygoumenitsa, Thesprotia, Greece). Voucher specimens are kept at the herbarium of the OPENSCREEN-GR infrastructure, at the University of Ioannina-Greece. The plant material was dried by freeze-drying process (lyophilization) and pulverized using an appropriate mixing and milling equipment (Polymix, Kinematica, Bohemia, NY, USA) to particles less than 2 mm. The Protective Geographical Indication (PGI) packaged olive oil “Lianoelia Prevezas” was provided by the olive oil mills of “Zalongo” and the extract of the Protective Geographical Indication (PGI) olives “Konservoelia Artas” olive oil was provided by a local olive mill (municipality Nikolaos Skoufas, Arta, Greece), while a sample of olive oil derived by the variety Lianoelia Kerkyras, grown in the region of Parga (municipality of Parga, Preveza, Greece), was provided by a producer.

### 4.3. Preparation of Infused Olive Oils

The infused olive oils were prepared by natural maceration for 30 days. In more detail, the dried and ground aromatic plant (0.25 g) was added to the olive oil sample (5 mL) in a screw-capped glass tube and was vigorously agitated for 1 min and kept for one month in the dark at room temperature. Olive oil samples without additives (untreated olive oils), stored in the same conditions, were used as control samples. After the extraction period, the enriched olive oils were obtained by centrifugation (2700× *g*, 25 min).

### 4.4. Preparation of Extracts

#### 4.4.1. Aromatic Plant Extracts

The pulverized dry plant material was extracted with methanol (10 mg mL^−1^) for 10 min × 3 using an ultrasound bath (Badelin Electronic, Berlin, Germany) as previously described [34], aliquoted and stored at −20 °C until use. The essential oil was isolated by hydro-distillation (2 h) using a Clevenger-type apparatus. Subsequently, the obtained essential oil was dried over anhydrous sodium sulfate (NaSO_4_) and stored at −20 °C until use.

#### 4.4.2. Extraction of Untreated and Infused Olive Oils

Liquid–liquid extraction using MeOH-H_2_O (80:20, *v*/*v*) as solvent was applied in order to receive the phenolic fraction from the olive oil’s samples according to the International Olive Council method (2009) [35]. In brief, 2.0 g of olive oil and 5 mL of MeOH-H_2_O (80:20, *v*/*v*) were mixed in a screw-capped glass tube under agitation for 1 min. Subsequently, the tube was sonicated with an ultrasonic bath (3 × 5 min) and the MeOH-H_2_O phase was recovered by centrifugation (2700× *g*, 25 min).

### 4.5. Phytochemical Analysis

#### 4.5.1. GC-MS Analysis of the Essential Oil

The essential oils, isolated from the aromatic plants, were analyzed by a gas chromatograph (GC) coupled with a mass spectrometer (MS) (GC-2030, GCMS-QPSERIES, Shimadzu, Kyoto, Japan). The essential oil constituents were separated using a Mega 5-MS capillary column (30 m × 0.25 mm × 0.25 μm), helium as carrier gas with a flow rate of 0.9 mL min^−1^ and a temperature program, previously described [36]. For the analysis, 1 μL of the essential oil was diluted in n-hexane (1:200) and then injected by autosampler (AOC-20i/s Shimadzu), while the injector was set at split mode (split ratio: 30). Each sample was analyzed twice. The injector, interface and ion source temperatures were set at 250 °C, 300 °C and 240 °C, respectively, while the mass spectrometer was operated at the electron ionization mode (70 eV), with the mass scan ranging from 50 to 550 amu, with 0.5 spectra s^−1^ acquisition rate. The compounds were identified based on the similarity of the acquired mass spectra with the Nist Library data and the comparison of their retention indices (RIs) relative to n-alkanes (C8–C20), with data obtained through the literature. The contribution of each identified ingredient to the essential oil was calculated as a percentage (%) of the total compounds.

#### 4.5.2. LC-LTQ/Orbitrap HRMS Analysis

An ultra-high-performance liquid chromatography (UHPLC) system coupled to a linear trap quadrupole (LTQ)/Orbitrap HRMS detector was employed for the identification of the phenolic compounds in all extracts. It was equipped with an autosampler (Accela AS autosampler model 2.1.1), an automatic sample flow pump (Accela quaternary gradient U-HPLC-pump model 1.05.0900) and a hybrid LTQ/Orbitrap XL 2.5.5 SP1 mass spectrometer from Thermo Fisher Scientific (Bremen, Germany). An Ion Max electrospray ionization (ESI) probe was included in the system. Compounds’ separation was performed on a reversed-phase Hypersil GOLD analytical column (100 mm × 2.1 mm, 1.9 μm) from Thermo (Bremen, Germany). The mobile phase consisted of a phase A (0.1% formic acid in water) and phase B (methanol), following a gradient elution program. Flow rate was set at 0.4 mL min^−1^. The injection volume was 10 μL, while the tray and oven temperatures were set at 15 and 40 °C, respectively. The system operated in negative ionization mode at a mass range of 100–1000 *m*/*z*. The ESI source conditions were: 55 and 20 arbitrary units (au) of sheath gas and aux gas flow rates, respectively; 350 °C capillary temperature; spray voltage, 2.7 kV. Full-scan mass spectra of high resolution were acquired in the Orbitrap analyzer with data-dependent MS/MS mode with parallel acquisition of top 6 intense ions scanned in the linear ion trap. A normalized collision energy (NCE) of 35% was used throughout the analysis (CID, collision induced dissociation) to obtain the compounds’ fragmentation pattern. The phenolic compounds were identified on the basis of their molecular ion formation and their characteristic fragments were compared to either the existing literature or to the NIST Mass Spectral Library 2020. The instruments’ control and the mass spectra processing were carried out with Xcalibur v.2.2 software (Thermo Electron, San Jose, CA, USA).

### 4.6. Free Radical Scavenging Capacity Assay

The determination of the free radical scavenging activity (RSA) of the aromatic plant extracts and the essential oil samples was performed according to Conforti et al. (2008) [37], after adaptation. Initially, the extract or the essential oil was diluted in methanol at concentrations ranging from 0.5 to 6.0 mg mL^−1^ for the essential oils and 0.05 to 0.40 mg mL^−1^ for the MeOH extracts. Subsequently, 100 μL of the solutions was added to 900 μL DPPH solution (0.1 mM final concentration in DPPH), the mixture was vigorously agitated and the absorbance was measured at 517 nm after staying for 30 min in the dark. Regarding the untreated and infused olive oils, the DPPH assay was performed as described by Minioti et al. (2010) [38]. In brief, olive oil samples (20, 80, 120 or 180 mg) were added to a DPPH solution in ethyl acetate (0.1 mM final concentration in DPPH). The mixture was vigorously agitated and, after 1 h in the dark, the absorbance was measured against blank (ethyl acetate solution) at 515 nm. Standard curve of gallic acid, used as a positive control, was plotted for concentrations ranging from 0.5 to 5.0 mg/mL of gallic acid. The scavenging activity was calculated by the following formula, using the absorbance of the DPPH solution (without the oil sample) as control absorbance.

% *DPPH scavenging activity* = [(*control absorbance* − *sample absorbance*)/*control absorbance*] × 100

The concentration of the aromatic plant extracts, or the amount of the olive oil samples required to achieve 50% inhibition of the DPPH free radical activity (IC_50_), was calculated from a plot of percent inhibition (%) against the sample concentration. The total antioxidant capacity of the olive oil samples was expressed as mL^−1^ gallic acid equivalent (GAE) kg^−1^ olive oil using the gallic acid standard curve. The antioxidant activity index (AAI) for the aromatic plant extracts was calculated through the following equation (Scherer and Godoy, 2009) [22]:

*AAI* = *final concentration of DPPH µg mL*^−1^/*IC*_50_ *µg mL*^−1^.

### 4.7. Determination of the Total Phenolic Content in the Extracts

The total phenolic content of the extracts (aromatic plants, olive oil) was estimated by a colorimetric method using Folin–Ciocalteu (FC) reagent and gallic acid as reference compound, as described by Vasdekis et al. (2018) [39]. In brief, 100 μL of the extract and 100 μL of the FC reagent were added to glass tube containing 4.5 mL of H_2_O; then, it was agitated for 1 min and, after 3 min, 300 μL of saturated Na_2_CO_3_ solution was added. The mixture was kept for 2 h in the dark and then the absorbance was measured at 760 nm. For the gallic acid standard curve, concentrations from 0.5 to 30.0 mg L^−1^ were used. The amount of the total phenolic content was calculated using the gallic acid standard curve and was expressed as mg GAE kg^−1^ olive oil or as mg GAE g^−1^ dry weight of the aromatic plant.

### 4.8. Statistical Analysis

The results were analyzed by using the Statistical Package IBM SPSS 26. The values were expressed as mean ± standard deviation of at least 4 independent experiments. The data concerning the phenolic content and the antioxidant capacity of the samples were analyzed by Anova, while Duncan’s multiple range test was conducted, with the significance set at *p* < 0.05.

## 5. Conclusions

Aromatic herbs and especially *S. triloba* used for producing infused olive oils in this study increased the total phenolic compounds and antioxidant activity of the enriched olive oils. The phenolic compounds that migrate from the plant tissue and enrich the olive oils belong mainly to the classes of aglycones and diterpenes. The migration of bioactive compounds from the aromatic herbs to the olive oils depends on their polarity. Enriched olive oils present increased free radical scavenging activity, leading to health benefits; however, further investigation of organoleptic properties is required for their acceptability by consumers.

## Figures and Tables

**Table 1:** Chemical composition of essential oils analyzed by GC-MS ^a^.

S/N	RT (min)	RI_EXP_	RI_Lit_	Compound Name	Area Percent (%)			Mode of Identification
					*O. vulgare*	*R. officinalis*	*S. triloba*	
1	5.83	925	925	Tricyclene		0.2	0.2	MS, RI
2	5.86	926	926	α-Thujene			0.2	MS, RI
3	6.1	934	934	α-Pinene		12.3	5.1	MS, RI
4	6.62	952	952	Camphene		2.8	5.0	MS, RI
5	6.72	955	955	Thuja-2,4(10)-diene		0.6		MS, RI
6	6.72	955	955	Sabinene			0.1	MS, RI
7	7.26	973		Unknown		0.2		
8	7.46	980	980	1-Octen-3-ol	0.3			MS, RI
9	7.47	980	980	β-Pinene		1.1	5.4	MS, RI
10	7.69	988	988	3-Octanone	0.1	0.1		MS, RI
11	7.73	990	990	Myrcene	0.3	0.7	3.3	MS, RI
12	7.86	993	993	2,3-Dehydro-1,8-cineole			0.1	MS, RI
13	8.4	1010	1010	α-Phellandrene			0.1	MS, RI
14	8.45	1011	1012	δ-3-Carene		2.0		MS, RI
15	8.77	1019	1019	α-Terpinene	0.2	0.1	0.3	MS, RI
16	9.08	1027	1027	p-Cymene	3.1	2.0	1.0	MS, RI
17	9.21	1031	1031	Limonene	0.1	3.5	1.6	MS, RI
18	9.31	1033	1033	β-Phellandrene	0.1			MS, RI
19	9.37	1035	1035	1,8 cineole	0.1	9.8	44.2	MS, RI
20	10.31	1059	1059	γ-Terpinene	1.4	0.1	0.4	MS, RI
21	10.84	1073	1073	cis-Sabinene hydrate	0.5	0.2	0.2	MS, RI
22	11.37	1087	1087	α-Terpinolene		0.4	0.1	MS, RI
23	11.66	1094	1094	p-Cymenene		0.2		MS, RI
24	11.98	1103	1103	Linalool	0.1	3.5	0.3	MS, RI
25	12.1	1106	1104	trans-Sabinene hydrate	0.2	0.3	0.2	MS, RI
26	12.37	1112	1112	α-Thujone			2.5	MS, RI
27	12.85	1123	1123	β-Thujone			1.1	MS, RI
28	12.9	1124	1124	Fenchol		0.1		MS, RI
29	13.02	1127	1127	Chrysanthenone		0.8		MS, RI
30	13.09	1128	1128	cis-p-Menth-2-ene-1-ol			0.1	MS, RI
31	13.23	1132	1132	α-Campholenal		0.1		MS, RI
32	13.73	1143	1143	Sabinol			0.1	MS, RI
33	13.84	1146	1146	cis-Verbenol		0.6		MS, RI
34	14.01	1150	1150	trans-Verbenol		0.9		MS, RI
35	14.18	1154	1154	Camphor		13.2	9.5	MS, RI
36	14.72	1166	1166	trans-Pinocamphone		0.8	0.1	MS, RI
37	14.82	1168	1168	Pinocarvone		0.3		MS, RI
38	15.11	1176	1175	δ-Terpineol			1.0	MS
39	15.21	1177	1177	Borneol	0.2	11.7	1.9	MS, RI
40	15.42	1183	1182	cis-Pinocamphone		2.5	0.1	MS, RI
41	15.53	1185	1185	Terpinen-4-ol	0.4	0.9	0.7	MS, RI
42	15.86	1193	1193	p-Cymen-8-ol		0.3		MS, RI
43	16.2	1200	1200	α-Terpineol	0.1	2.4	3.3	MS, RI
44	16.4	1203	1203	trans-Dihydrocarvone	0.2			MS, RI
45	16.5	1205		Unknown		1.0		
46	16.81	1211	1211	Verbenone		11.5	0.1	MS, RI
47	17.24	1219	1219	cis-Carveol		0.1		MS, RI
48	18.33	1239	1239	cis-Shisool		0.9		MS, RI
49	18.58	1243	1243	Carvone		0.1		MS, RI
50	18.58	1244	1244	Linalyl acetate			0.1	MS, RI
51	18.69	1246	1246	trans-Shisool		2.0		MS, RI
52	19.04	1252	1252	cis-Myrtanol		0.1		MS, RI
53	19.58	1262	1262	trans-Myrtanol		0.1		MS, RI
54	20.01	1271	1271	Isopiperitenone		0.2		MS, RI
55	20.49	1278	1278	cis-Verbenyl acetate		0.1		MS, RI
56	20.75	1284	1284	Bornyl acetate		2.2	0.9	MS, RI
57	21.23	1292	1292	Thymol	4.6			MS, RI
58	21.74	1303	1303	Carvacrol	86.4	0.1		MS, RI
59	24.91	1339	1339	Piperitenone		0.2		MS, RI
60	25.41	1345	1345	α-Terpinyl acetate			1.2	MS, RI
61	27.73	1371	1371	Copaene		0.3		MS, RI
62	30.44	1405	1405	Methyleugenol		0.2		MS, RI
63	31.17	1416	1416	β-Caryophyllene	0.6	1.9	2.8	MS, RI
64	32.46	1436	1436	Aromandendrene		0.1	0.4	MS, RI
65	33.64	1454	1454	α-Humulene	0.1	0.5	0.8	MS, RI
66	33.9	1457	1457	Alloaromadendrene			0.1	MS, RI
67	34.96	1474	1474	γ-Muurolene		0.3		MS, RI
68	35.83	1487	1487	Viridiflorene			0.2	MS, RI
69	36.4	1495	1495	α-Muurolene		0.1		MS, RI
70	37.03	1507	1507	β-Bisabolene	0.5	0.1		MS, RI
71	37.2	1511	1511	γ-Cadinene		0.3		MS, RI
72	37.5	1518	1518	δ-Cadinene	0.1	0.5	0.1	MS, RI
73	37.63	1520	1520	cis-Calamenene		0.1		MS, RI
74	37.73	1523	1523	trans-Calamenene		0.2		MS, RI
75	38.76	1546	1546	α-Calacorene		0.1		MS, RI
76	40.43	1584		Unknown			0.2	
77	40.58	1588	1588	Caryophyllene oxide	0.5	1.0	1.0	MS, RI
78	40.7	1591	1591	Globulol			0.1	MS, RI
79	40.92	1596	1596	Viridiflorol			2.0	MS, RI
80	41.02	1597		Unknown			0.1	
81	41.04	1598	1598	Humulene epoxide I		0.1		MS, RI
82	41.27	1611	1611	Humulene epoxide II	0.1	0.2	0.3	MS, RI
83	41.59	1633	1633	Epicubenol		0.1		MS, RI
84	41.67	1637		Humulenol-II		0.2	0.1	MS
85	41.74	1646	1646	Caryophylla-4(12),8(13)-dien-5α-ol		0.2	0.1	MS, RI
86	41.82	1651	1651	τ-Cadinol		0.1	0.1	MS, RI
87	41.85	1652	1652	Cadin-4-en-10-ol		0.1		MS, RI
88	42.2	1679	1679	Germacra-4(15),5,10(14)-trien-1α -ol		0.2	0.4	MS, RI
89	42.37	1692	1692	α-Bisabolol		0.2		MS, RI
90	44.76			Manool			0.8	MS
**Monoterpene hydrocarbons**					5.1	26.2	22.8	
**Oxygenated monoterpens**					92.7	63.7	65.4	
**Sesquiterpene hydrocarbons**					1.3	4.5	4.4	
**Oxygenated sesquiterpenes**					0.6	2.4	4.1	
**Diterpenes**							0.8	
**Others**					0.5	2.6	2.2	
**Unkown**					0	1.2	0.3	
**Monoterpenes**					97.8	89.9	88.2	
**Sesquiterpene**					1.8	6.9	8.5	

^a^ The contribution of each identified ingredient to each essential oil was calculated as a percentage (%) of the total identified compounds in a representative GC-MS chromatogram. **RT**: retention time, **RI_EXP_**: experimentally determined retention index, **RI_Lit_**: retention index, according to NIST Chemistry Web Book, SRD 69.

**Table 2:** Phenolic compounds in the extracts ^#^ of *O. vulgare* ssp. *hirtum*, and the relevant enriched olive oils, as identified by LC-LTQ/Orbitrap HRMS.

Identified Compounds	ESI	Ion Form	Theoretical *m*/*z*	Mass Error (ppm)	MS/MS Fragments	Molecular Formula	Aromatic Herbs MeOH Extracts	Hydro-Methanolic Extracts of Olive Oils		
								LP	KA	LK
Salicylic acid	-	[Μ − H]^−^	137.024	1.779	**93**	C_7_H_6_O_3_	√	√	√	√
Caffeic acid hexoside	-	[Μ − H]^−^	341.087	1.421	**179.05**/161.04/135.13 *	C_15_H_18_O_9_	√			
Luteolin-6,8-di-c-hexose	-	[Μ − H]^−^	609.146	1.021	**489.10**/519.11/399.11 **	C_27_H_30_O_16_	√			
Apigenin 6,8-di-C-glucoside	-	[Μ − H]^−^	593.15	2.303	**473.10**/353.11/503.10 ***	C_27_H_30_O_15_	√			
Luteolin-6-C-glucoside	-	[Μ − H]^−^	447.093	1.622	**327.08**/357.07/429.06	C_21_H_20_O_11_	√			
Rosmarinic acid	-	[Μ − H]^−^	359.077	1.986	**161.02**/197.05/179.02	C_18_H_16_O_8_	√			
Rosmarinic acid	-	[Μ − H]^−^	359.077	1.776	**161.03**/197.04/179.04	C_18_H_16_O_8_	√			
Apigenin 8-C-glucoside	-	[Μ − H]^−^	431.098	1.447	**311.03**/341.07 ***	C_21_H_20_O_10_	√			
Taxifolin	-	[Μ − H]^−^	303.005	1.281	**285.05**/177.08/125.07	C_15_H_12_O_7_	√	√	√	√
Eriodictyol 7-O-glucoside	-	[Μ − H]^−^	449.109	2.022	**287.06**	C_21_H_22_O_11_	√			
Dihydrokaempferol	-	[Μ − H]^−^	287.056	1.475	**259.06**/243.10/269.06 ***	C_15_H_12_O_6_	√			
Eridictyol **	-	[Μ − H]^−^	287.056	0.657	**151.01**/125.09/135.04	C_15_H_11_O_6_	√	√	√	√
Salvianolic acid B	-	[Μ − H]^−^	717.148	2.699	519.06/321.15/339.17	C_36_H_30_O_16_				
Luteolin	-	[Μ − H]^−^	285.039	1.806	**241.02**/175.11/199.06	C_15_H_10_O_6_	√	√	√	√
Apigenin	-	[Μ − H]^−^	269.046	2.120	**225.07**/149.00/201.05	C_15_H_10_O_5_	√	√	√	√
Naringenin	+	[Μ + H]^+^	273.0758	1.550	152.97/147.03	C_15_H_12_O_5_	√	√	√	√
Acacetin	-	[Μ − H]^−^	283.0601	1.790	**268.09**/283.16/239.22	C_16_H_12_O_5_	√	√	√	√

^#^ *O. vulgare* was extracted with methanol, while the relevant enriched olive oils were extracted with [MeOH-H_2_O (80:20, *v*/*v*)]. √: signifies the presence of the relevant compound. Bold numbers represent the most abundant MS/MS fragment; LP: “Lianoelia Prevezas”; KA: “Konservoelia Artas”; LK: “Lianoelia Kerkyras” olive oils. * Castañeta et al., 2022 [6]. ** Geng et al., 2016 [7]. *** Dias et al., 2013 [8].

**Table 3:** Phenolic compounds in the extracts ^#^ of *R. officinalis* and the relevant enriched olive oils, as identified by LC-LTQ/Orbitrap HRMS.

Identified Compounds	ESI	Ion Form	Theoretical *m*/*z*	Mass Error (ppm)	MS/MS Fragments	Molecular Formula	Aromatic Herb MeOH Extract	Hydro-Methanolic Extracts of Olive Oils		
								LP	KA	LK
Rosmarinic acid	-	[Μ − H]^−^	359.0772	1.006	**161.03**/197.05/179.03	C_18_H_16_O_8_	√			
Caffeic acid	-	[Μ − H]^−^	179.0339	1.335	**135.03**	C_9_H_8_O_4_	√			
Sagerinic acid	-	[Μ − H]^−^	719.1618	1.389	359.01 *	C_36_H_32_O_16_	√			
Hesperidin	-	[Μ − H]^−^	609.1814	1.463	**301.04**	C_28_H_34_O_15_	√			
Rosmarinic acid	-	[Μ − H]^−^	359.0772	1.306	**161.03**/197.04/179.04	C_18_H_16_O_8_	√			
Hispidulin-7-glucoside	-	[Μ − H]^−^	461.1078	0.714	299.06 **	C_22_H_22_O_11_	√			
Apigenin	-	[Μ − H]^−^	269.0445	1.02	**225.06**/149.02/201.06	C_15_H_10_O_5_	√	√	√	√
Pectolinarigenin	-	[Μ − H]^−^	313.0707	1.295	**298.05/283.02**	C_17_H_14_O_6_	√	√	√	√
Rosmanol	-	[Μ − H]^−^	345.1697	1.65	**301.19** **	C_20_H_26_O_5_	√	√	√	√
Hydroxyrosmanol	-	[Μ − H]^−^	361.1646	1.385	317.18 **	C_20_H_26_O_6_	√	√	√	√
Genkwanin	-	[Μ − H]^−^	283.0601	1.24	**268.04**/283.06	C_16_H_12_O_5_	√	√	√	√
Rosmanol methyl ether	-	[Μ − H]^−^	359.1853	0.77	**283.17**/329.18/300.17 **	C_21_H_28_O_5_	√	√	√	√
Rosmadial	-	[Μ − H]^−^	343.1540	1.43	**315.14**/299.17/287.17 **	C_20_H_24_O_5_	√	√	√	√
Rosmaridiphenol	-	[Μ − H]^−^	315.1955	1.579	285.19 **	C_20_H_28_O_3_	√	√	√	√
Carnosol	+	[Μ + H]^+^	331.1904	1.174	**285.05**/289.11/303.06	C_20_H_26_O_4_	√	√	√	√
Carnosol	-	[Μ − H]^−^	329.1747	1.654	**285.19** **	C_20_H_26_O_4_ **	√	√	√	√
Carnosic acid	-	[Μ − H]^−^	331.1904	1.413	287.20/244.15 **	C_20_H_28_O_4_	√	√	√	√

^#^ *R. officinalis* was extracted with methanol, while the relevant enriched olive oils were extracted with [MeOH-H_2_O (80:20, *v*/*v*)]. √: signifies the presence of the relevant compound. Bold numbers represent the most abundant MS/MS fragment. LP: “Lianoelia Prevezas”, KA: “Konservoelia Artas”, LK: “Lianoelia Kerkyras” olive oils. * Sharma et al., 2020 [9]. ** Castañeta et al., 2022 [6].

**Table 4:** Phenolic compounds in the extracts ^#^ of *S. triloba* and the respective enriched olive oils, as identified by LC-LTQ/Orbitrap HRMS.

Identified Compounds	ESI	Ion Form	Theoretical *m*/*z*	Mass Error (ppm)	MS/MS Fragments	Molecular Formula	Aromatic Herb MeOH Extract	Hydro-Methanolic Extracts of Olive Oils		
								LP	KA	LK
Vanillic acid	-	[Μ − H]^−^	167.0350	1.535	**123.06**/152.03/108.09	C_8_H_8_O_4_	√	√		√
Caffeic acid	-	[Μ − H]^−^	179.0339	1.375	135.03	C_9_H_8_O_4_	√			
Nepetrin	-	[Μ − H]^−^	477.1033	0.948	**315.04**/300.05/461.97	C_22_H_22_O_12_	√			
Rosmarinic acid	-	[Μ − H]^−^	359.0767	1.526	**161.03**/197.02/179.03	C_18_H_16_O_8_	√			
Sagerinic acid	-	[Μ − H]^−^	719.1618	2.179	359.01 *	C_36_H_32_O_16_	√			
Luteolin-7-O-glucoside	-	[Μ − H]^−^	447.0927	1.742	285.05 **	C_21_H_20_O_11_	√			
Luteolin-3-O-glucuronide	-	[Μ − H]^−^	461.0720	1.382	285.03 **	C_21_H_18_O_12_	√			
Quercetin	-	[Μ − H]^−^	301.0354	0.753	**178.99**/151.03/273.01	C_15_H_10_O_7_	√	√	√	√
Nepetin	-	[Μ − H]^−^	315.0510	1.551	**300.03**/297.15	C_16_H_12_O_7_	√			
Rosmanol	-	[Μ − H]^−^	345.1702	1.31	**301.19**	C_20_H_26_O_5_	√	√	√	√
Apigenin	-	[Μ − H]^−^	269.0455	1.02	**225.08**/149.13/201.08	C_15_H_10_O_5_	√	√	√	√
Hydroxyrosmanol	-	[Μ − H]^−^	361.1615	1.385	**317.18**	C_20_H_26_O_6_	√	√	√	√
Pectolinarigenin	-	[Μ − H]^−^	313.0718	1.755	**298.00**/283.06	C_17_H_14_O_6_	√	√	√	√
Rosmanol methyl ether	-	[Μ − H]^−^	359.1858	1.530	283.16 **	C_21_H_28_O_5_	√	√	√	√
Rosmadial	-	[Μ − H]^−^	343.1545	0.910	**315.14**/299.17/287.17 **	C_20_H_24_O_5_	√	√	√	√
Rosmaridiphenol	-	[Μ − H]^−^	315.1960	1.089	**285.20** **	C_20_H_28_O_3_	√	√	√	√
Carnosol	+	[Μ + H]^+^	331.1904	1.174	**285.05**/289.11/303.06	C_20_H_26_O_4_	√	√	√	√
Carnosol	-	[Μ − H]^−^	329.1747	1.194	**285.19** **	C_20_H_26_O_4_	√	√	√	√
Carnosic acid	-	[Μ − H]^−^	331.1909		**287.24**/244.22 **	C_20_H_28_O_4_	√	√	√	√

^#^ *S. triloba* was extracted with methanol, while the relevant enriched olive oils were extracted with [MeOH-H_2_O (80:20, *v*/*v*)]. √: signifies the presence of the relevant compound. Bold numbers represent the most abundant MS/MS fragment of the relevant identified compound. LP: “Lianoelia Prevezas”, KA: “Konservoelia Artas”, LK: “Lianoelia Kerkyras” olive oils. * Sharma et al., 2020 [9]. ** Castañeta et al., 2022 [6].

**Table 5:** Antioxidant activity of the untreated and infused olive oils as determined by DPPH assay. The mean values ± SD of IC_50_ values are expressed as mg oil and as mM GAE/Kg olive oil. Different letters within the same column state that the mean values present statistically significant differences (Duncan’s test, *p* < 0.05).

	Olive Oil (mg)	Olive Oil (mM GAE/Kg)
	**Lianoelia Prevezas**	
Olive oil	76.3 ± 0.2 d	185.8 ± 4.1 d
Olive oil + *O. vulgare* ssp. *hirtum*	67.5 ± 1.3 c	210.0 ± 4.1 c
Olive oil + *R. officinalis*	50.4 ± 5.2 b	283.4 ± 29.0 b
Olive oil + *S. triloba*	22.1 ± 0.3 a	641.4 ± 6.7 a
	**Konservoelia Artas**	
Olive oil	106.8 ± 2.1 d	132.6 ± 2.61.0 d
Olive oil + *O. vulgare* ssp. *hirtum*	84.7 ± 1.6 c	167.3 ± 3.2 c
Olive oil + *R. officinalis*	65.4 ± 1.7 b	216.7 ± 5.7 b
Olive oil + *S. triloba*	26.4 ± 0.3 a	535.9 ± 6.8 a
	**Lianoelia Kerkyras**	
Olive oil	83.7 ± 1.6 d	169.4 ± 3.3 d
Olive oil + *O. vulgare* ssp. *hirtum*	58.7 ± 0.6 c	241.4 ± 2.6 c
Olive oil + *R. officinalis*	41.4 ± 0.5 b	342.1 ± 4.0 b
Olive oil + *S. triloba*	21.1 ± 0.7 a	672.6 ± 22.2 a

## Data Availability

The data presented in this study are available in the article and Supplementary Materials.

## Supplementary Materials

The following supporting information can be downloaded at: https://www.mdpi.com/article/10.3390/molecules29051141/s1, Figure S1: GC-MS chromatograms, Figure S2: LC-LTQ/Orbitrap HRMS Chromatograms.
