# Supplementary material for: Enrichment of Olive Oils with Natural Bioactive Compounds from Aromatic and Medicinal Herbs: Phytochemical Analysis and Antioxidant Potential
Source: Molecules. 2024 Mar 4;29(5):1141. doi: 10.3390/molecules29051141 (PMC10934313; doi:10.3390/molecules29051141)
Supplement: Supplementary file 1 [file molecules-29-01141-s001.zip › Figure S1.pdf]

Figure S1-A: GC-MS Chromatogram of *O. Vulgare ssp. hirtum* essential oil

Figure S1-B: GC-MS Chromatogram of *Rosmarinus officinalis* essential oil

Figure S1-C: GC-MS Chromatogram of *Salvia triloba* essential oil

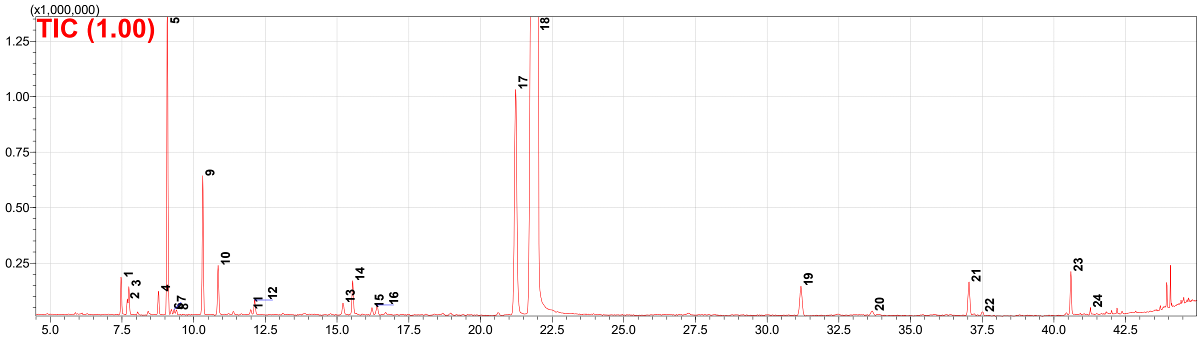

Figure S1-A. Chromatogram of *O. Vulgare ssp. hirtum* essential oil

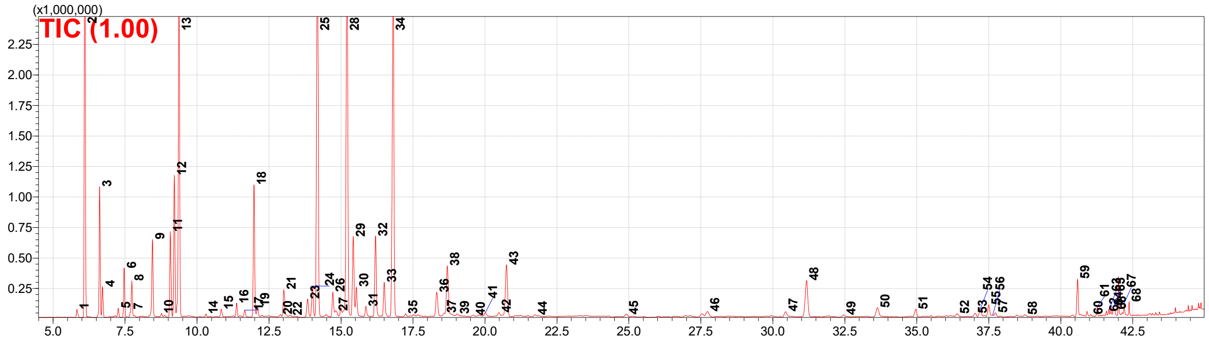

Figure S1-B: GC-MS Chromatogram of *Rosmarinus officinalis* essential oil

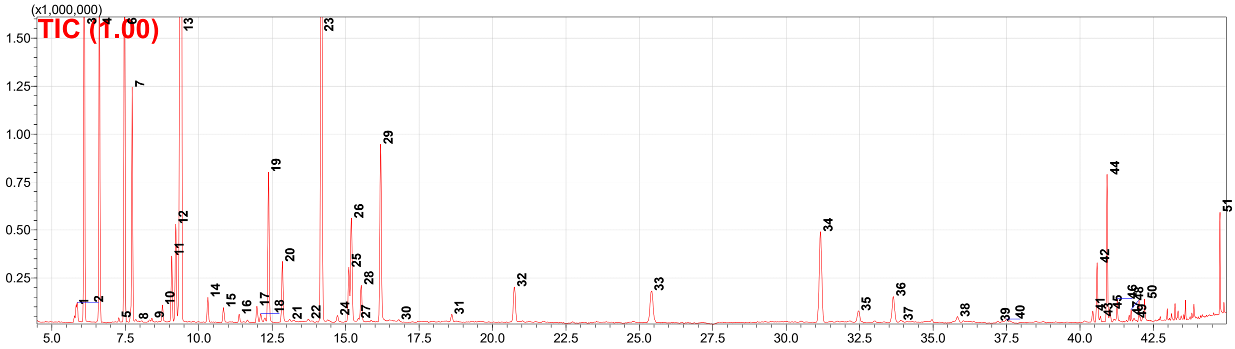

Figure S1-C: GC-MS Chromatogram of *Salvia triloba* essential oil
