# Supplementary material for: Enrichment of Olive Oils with Natural Bioactive Compounds from Aromatic and Medicinal Herbs: Phytochemical Analysis and Antioxidant Potential
Source: Molecules. 2024 Mar 4;29(5):1141. doi: 10.3390/molecules29051141 (PMC10934313; doi:10.3390/molecules29051141)
Supplement: Supplementary file 1 [file molecules-29-01141-s001.zip › Figure S2.pdf]

Figure S2-A: LTQ/Orbitrap HRMS Chromatogram of *Origanum vulgare* ssp *hirtum* methanol extract.

Figure S2-B: LTQ/Orbitrap HRMS Chromatogram of *Rosmarinus officinalis* methanol extract.

Figure S2-C: LTQ/Orbitrap HRMS Chromatogram of *Salvia triloba* methanol extract.

Figure S2-D: LTQ/Orbitrap HRMS Chromatogram of hydro-methanolic extracts [MeOH-H_2O (80:20, v/v)] of the enriched "Lianolia Prevezas" olive oil with *Origanum vulgare* ssp *hirtum.*

Figure S2-E: LTQ/Orbitrap HRMS Chromatogram of hydro-methanolic extracts [MeOH-H_2O (80:20, v/v)] of the enriched "Lianolia Prevezas" olive oil with *Rosmarinus officinalis.*

Figure S2-F: LTQ/Orbitrap HRMS Chromatogram of hydro-methanolic extracts [MeOH-H_2O (80:20, v/v)] of the enriched "Lianolia Prevezas" olive oil with *Salvia triloba.*

Figure S2-G: LTQ/Orbitrap HRMS Chromatogram of hydro-methanolic extracts [MeOH-H_2O (80:20, v/v)] of the enriched "Konservoelia Artas " olive oil with *Origanum vulgare* ssp *hirtum.*

Figure S2-H: LTQ/Orbitrap HRMS Chromatogram of hydro-methanolic extracts [MeOH-H_2O (80:20, v/v)] of the enriched "Konservoelia Artas " olive oil with *Rosmarinus officinalis.*

Figure S2-I: LTQ/Orbitrap HRMS Chromatogram of hydro-methanolic extracts [MeOH-H_2O (80:20, v/v)] of the enriched "Konservoelia Artas " olive oil with *Salvia triloba.*

Figure S2-J: LTQ/Orbitrap HRMS Chromatogram of hydro-methanolic extracts [MeOH-H_2O (80:20, v/v)] of the enriched "Lianoelia Kerkyras" olive oil with *Origanum vulgare* ssp *hirtum.*

Figure S2-K: LTQ/Orbitrap HRMS Chromatogram of hydro-methanolic extracts [MeOH-H_2O (80:20, v/v)] of the enriched "Lianoelia Kerkyras" olive oil with *Rosmarinus officinalis.*

Figure S2-L: LTQ/Orbitrap HRMS Chromatogram of hydro-methanolic extracts [MeOH-H_2O (80:20, v/v)] of the enriched "Lianoelia Kerkyras" olive oil with *Salvia triloba.*

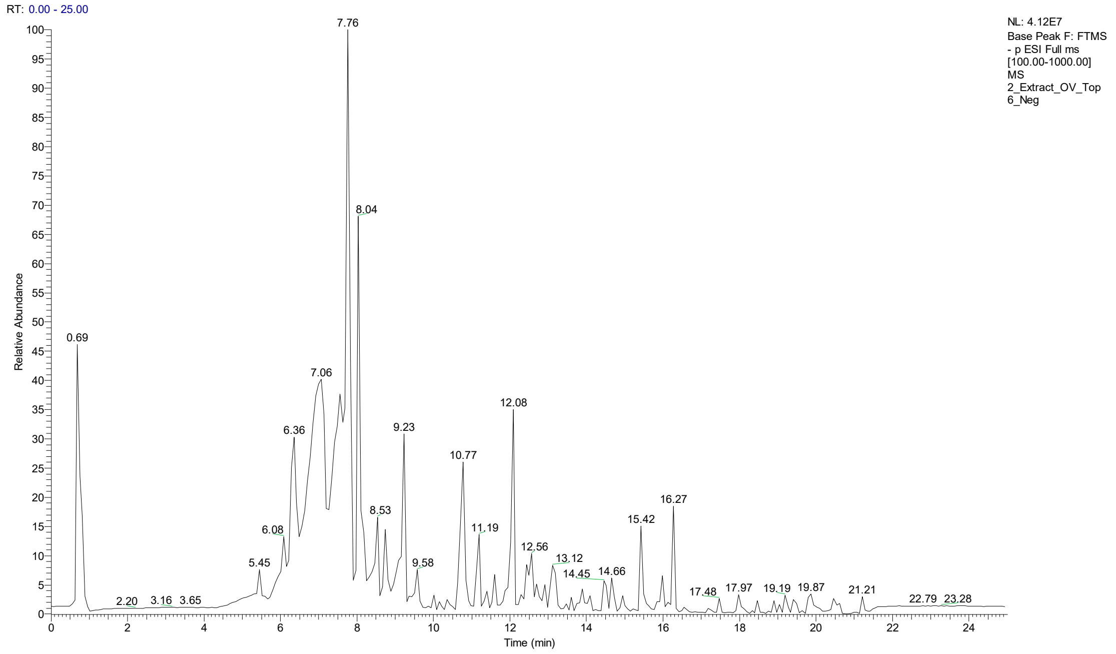

Figure S2-A: LTQ/Orbitrap HRMS Chromatogram of *Origanum vulgare* ssp *hirtum* methanol extract in negative ion mode.

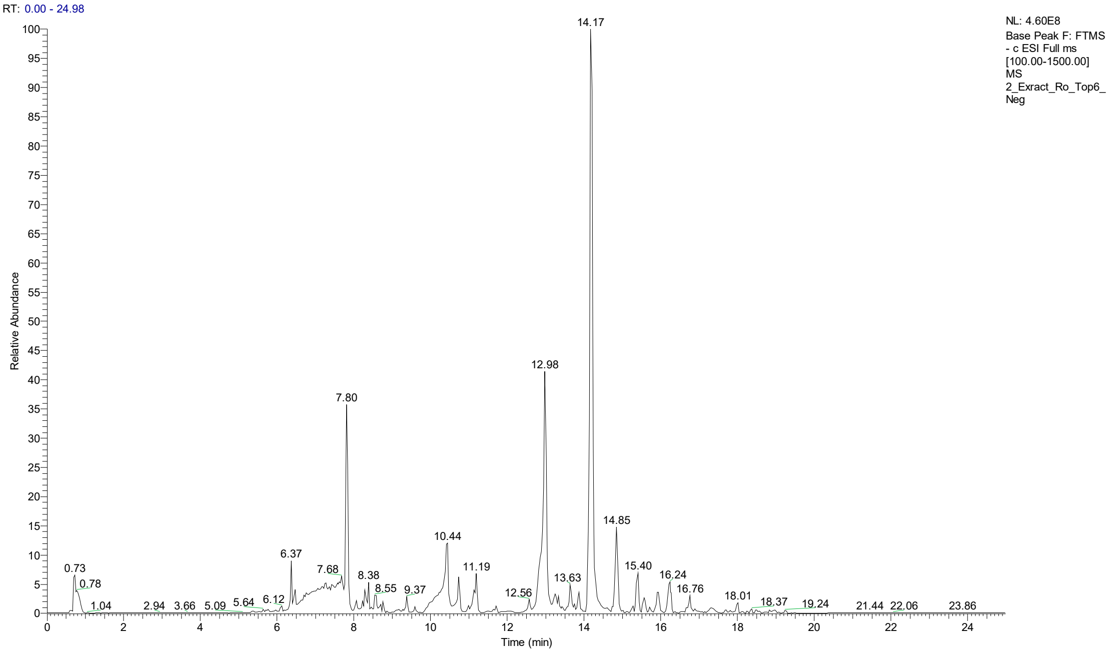

Figure S2-B: LTQ/Orbitrap HRMS Chromatogram of *Rosmarinus officinalis* methanol extract in negative ion mode.

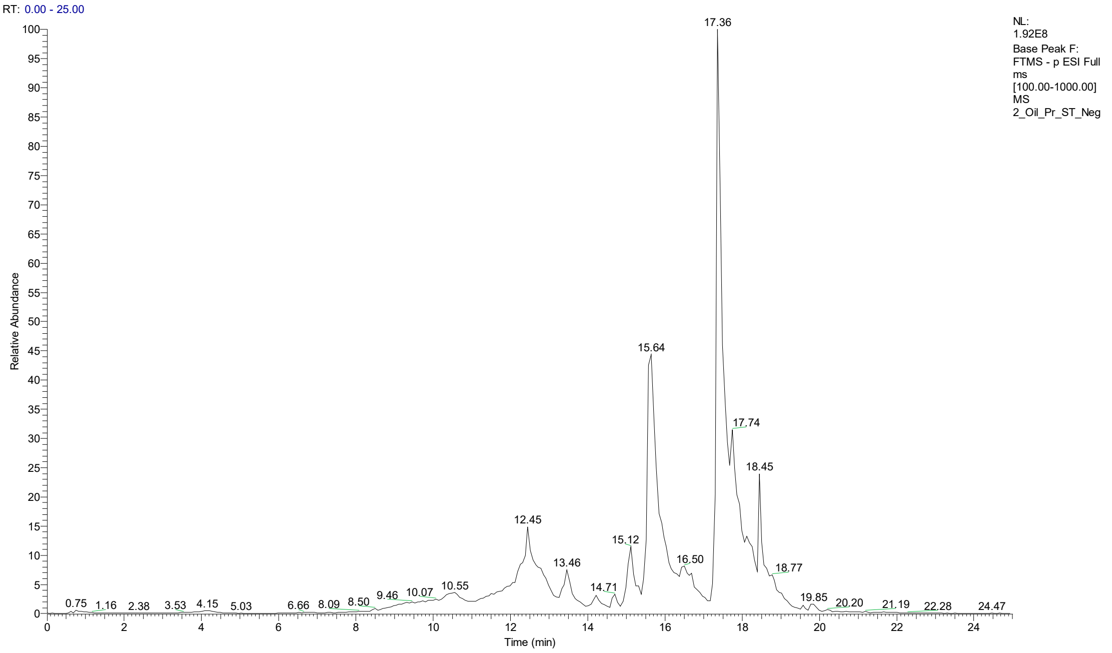

Figure S2-C: LTQ/Orbitrap HRMS Chromatogram of *Salvia triloba* methanol extract in negative ion mode.

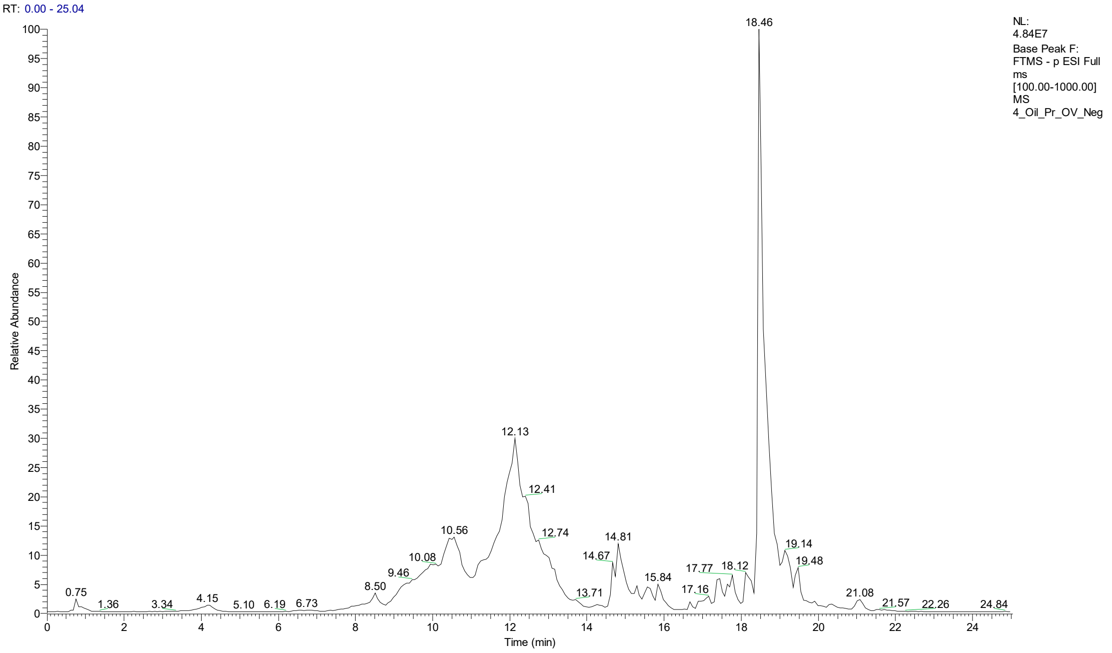

Figure S2-D: LTQ/Orbitrap HRMS Chromatogram of hydro-methanolic extracts [MeOH-H_2O (80:20, v/v)] of the enriched "Lianolia Prevezas" olive oil with *Origanum vulgare* ssp *hirtum* in negative ion mode.

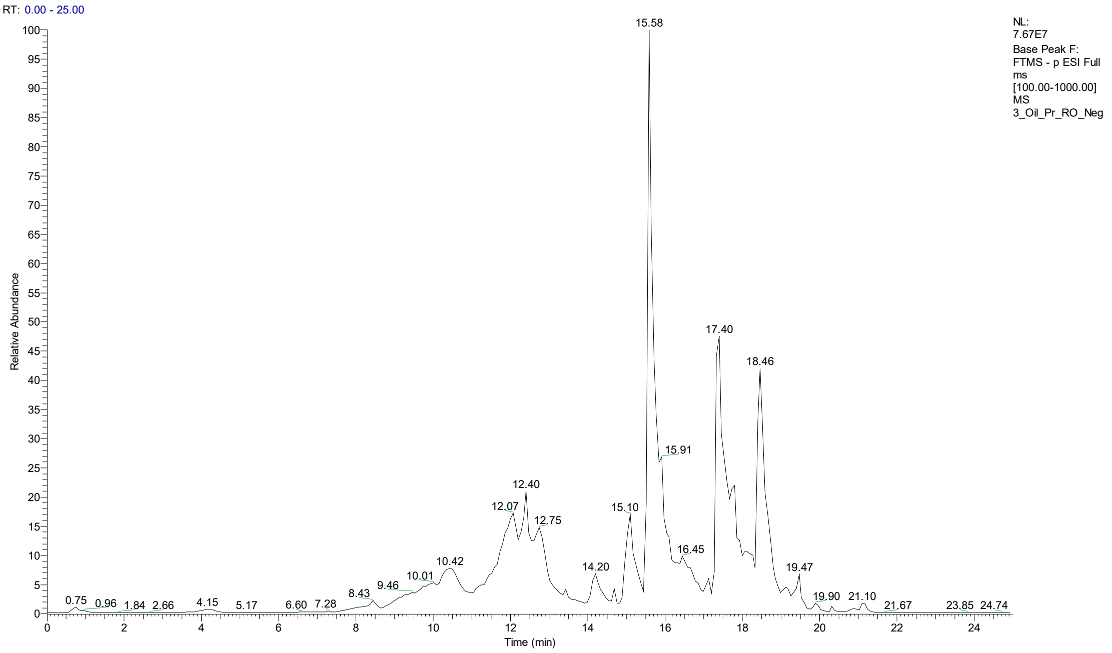

Figure S2-E: LTQ/Orbitrap HRMS Chromatogram of hydro-methanolic extracts (MeOH 80/20, v/v) of the enriched "Lianolia Prevezas" olive oil with *Rosmarinus officinalis* in negative ion mode.

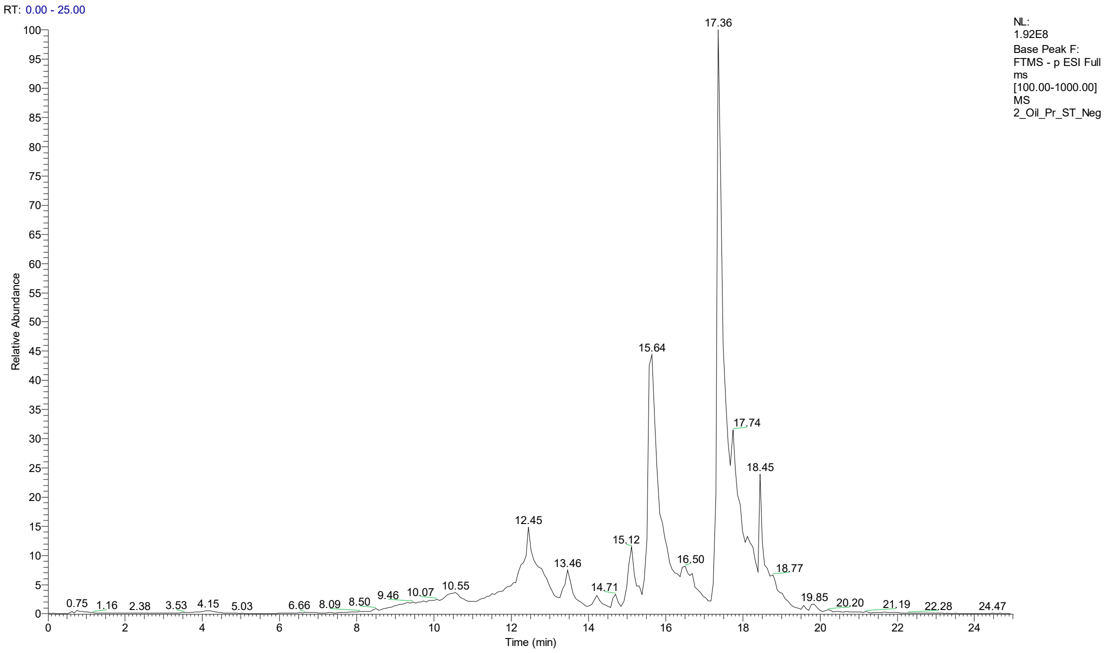

Figure S2-F: LTQ/Orbitrap HRMS Chromatogram of hydro-methanolic extracts [MeOH-H_2O (80:20, v/v)] of the enriched "Lianolia Prevezas" olive oil with *Salvia triloba* in negative ion mode.

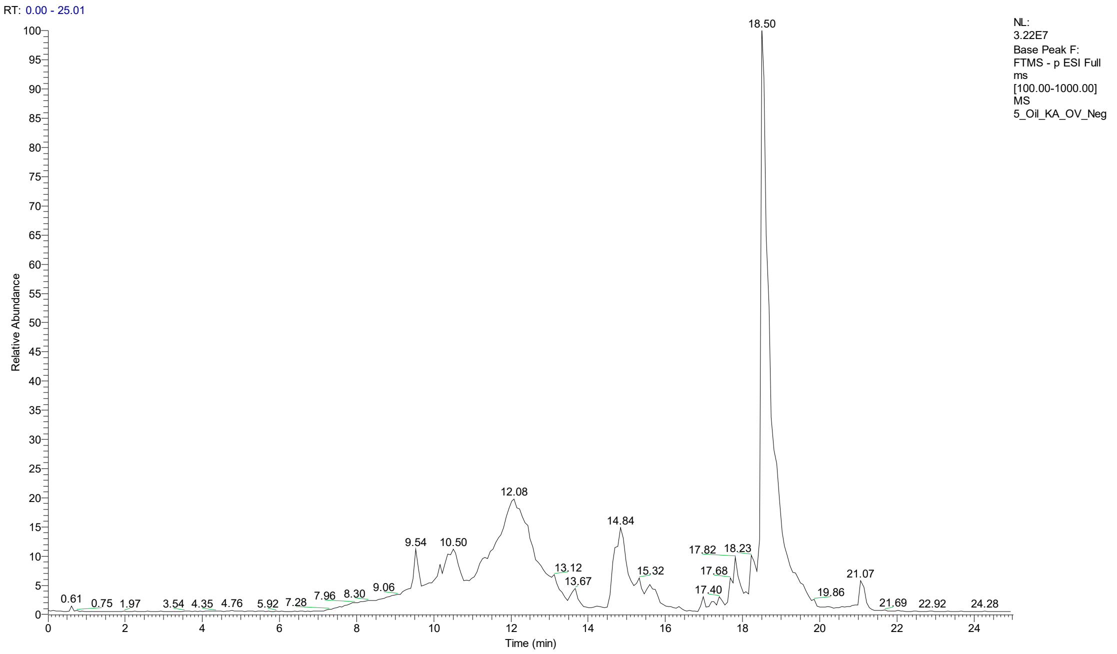

Figure S2-G: LTQ/Orbitrap HRMS Chromatogram of hydro-methanolic extracts [MeOH-H_2O (80:20, v/v)] of the enriched "Konservoelia Artas " olive oil with *Origanum vulgare* ssp *hirtum* in negative ion mode.

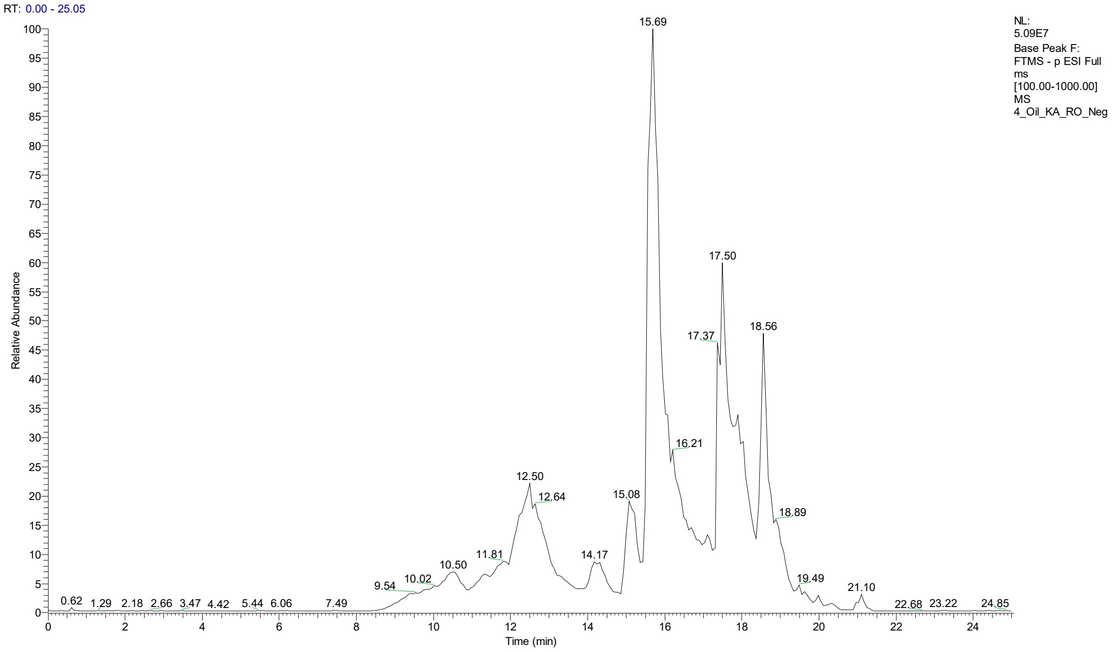

Figure S2-H: LTQ/Orbitrap HRMS Chromatogram of hydro-methanolic extracts (MeOH 80/20, v/v) of the enriched "Konservoelia Artas " olive oil with *Rosmarinus officinalis* in negative ion mode.

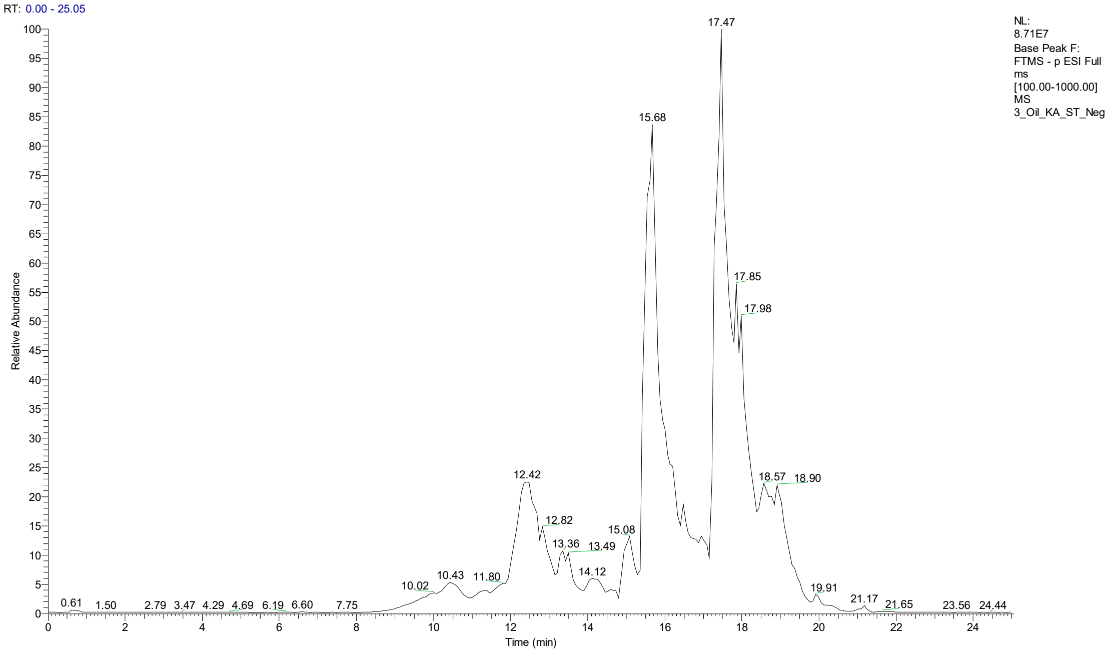

Figure S2-I: LTQ/Orbitrap HRMS Chromatogram of hydro-methanolic [MeOH-H_2O (80:20, v/v)] of the enriched "Konservoelia Artas " olive oil with *Salvia triloba* in negative ion mode.

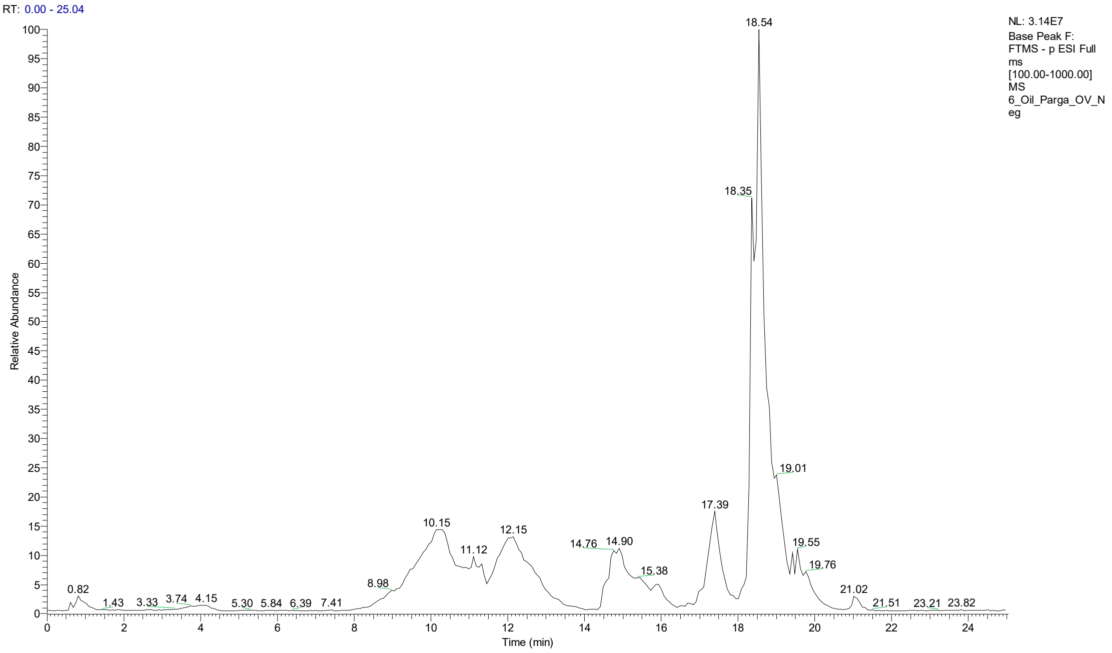

Figure S2-J: LTQ/Orbitrap HRMS Chromatogram of hydro-methanolic extracts [MeOH-H_2O (80:20, v/v)] of the enriched "Lianoelia Kerkyras" olive oil with *Origanum vulgare* ssp *hirtum* in negative ion mode.

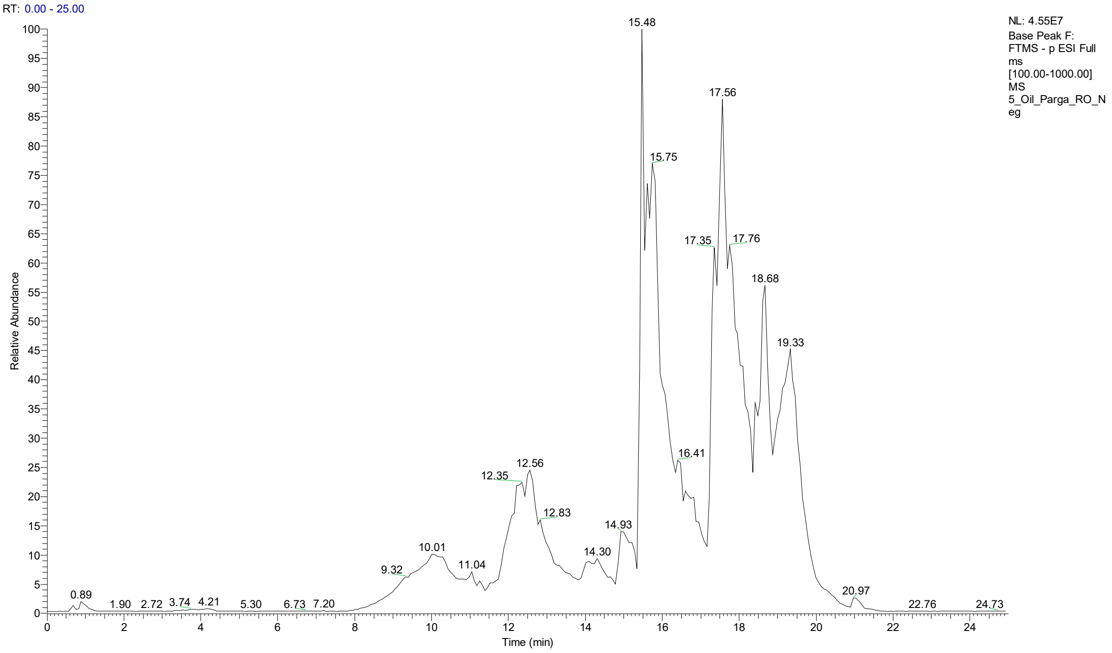

Figure S2-K: LTQ/Orbitrap HRMS Chromatogram of hydro-methanolic extracts [MeOH-H_2O (80:20, v/v)] of the enriched "Lianoelia Kerkyras" olive oil with *Rosmarinus officinalis* in negative ion mode.

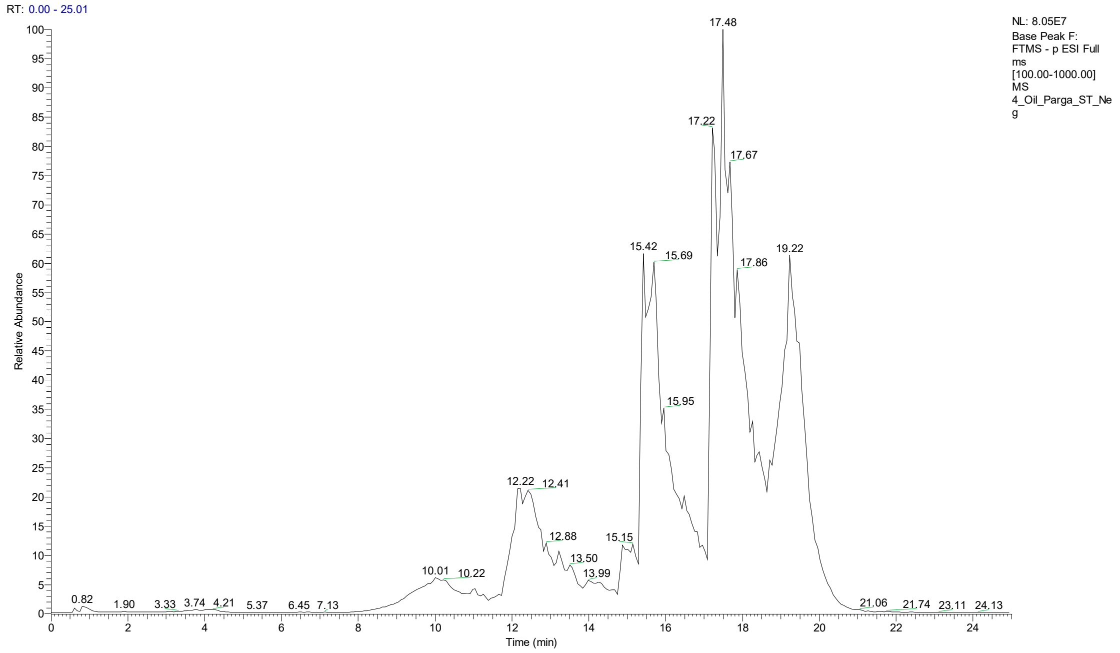

Figure S2-L: LTQ/Orbitrap HRMS Chromatogram of hydro-methanolic extracts [MeOH-H_2O (80:20, v/v)] of the enriched "Lianoelia Kerkyras" olive oil with *Salvia triloba* in negative ion mode.
